# Effectiveness of Psychotherapy for Post-Traumatic Stress Disorder in Subjects Suffering from Traumatic Brain Injuries After Motor Vehicle Accidents

**DOI:** 10.3390/healthcare13101194

**Published:** 2025-05-20

**Authors:** Agnieszka Popiel, Beata Banaszak, Ewa Pragłowska, Bogdan Zawadzki

**Affiliations:** 1 ACSTEC—Advanced Clinical Studies and Therapy Excellence Center, SWPS University, 03-815 Warsaw, Poland; 2 Clinic of Cognitive-Behavioral Therapy, SWPS University, 03-815 Warsaw, Poland; bbanaszak@swps.edu.pl (B.B.); epraglowska@swps.edu.pl (E.P.); 3 Faculty of Psychology, University of Warsaw, 00-183 Warsaw, Poland

**Keywords:** post-traumatic stress disorder (PTSD), traumatic brain injury (TBI), effectiveness, psychotherapy, prolonged exposure, motor vehicle accident (MVA)

## Abstract

**Background and Objectives:** PTSD and traumatic brain injury (TBI) frequently co-occur in survivors of combat exposure, blasts, assaults, or motor vehicle accidents (MVAs), yet the impact of TBI on the psychotherapy outcomes for PTSD, especially in civilians, remains underexplored and frequently underestimated. **Methods**: This study focused on analysis of the effectiveness of psychotherapies (trauma-focused: prolonged exposure (PE); non-trauma-focused: self-efficacy-focused cognitive therapy (SEF-CT)) in individuals with PTSD, comparing those with and without TBIs. The data of 45 PTSD patients with TBIs were drawn from a clinical trial cohort, with a total of 134 completing treatment. PTSD symptoms were assessed pre- and post-treatment using CAPS-5 and PDS-5. Cognitive functioning was measured via tests of fluid and crystallized intelligence. ANCOVA models examined the level of post-treatment PTSD symptoms with the control of pretreatment symptoms and the effects of TBI, treatment type, gender, age, education, time since the MVA, and level of cognitive functioning. **Results**: Both psychotherapies were equally effective in reducing PTSD symptoms, regardless of TBI status. The early initiation of treatment predicted better outcomes in non-TBI patients but not in those with TBIs. The TBI participants who began treatment earlier exhibited lower fluid intelligence scores, suggesting mild cognitive impairments that may have moderated the therapy benefits. **Conclusions**: Patients with PTSD and TBIs can benefit from both trauma-focused and non-trauma-focused CBT. While earlier intervention is beneficial for patients with PTSD alone, cognitive impairments may reduce this advantage in those with TBIs.

## 1. Introduction

Post-traumatic stress disorder (PTSD) is a complex psychiatric condition that develops following exposure to traumatic events such as violence, accidents, combat, or natural disasters. It is characterized by three (according to the WHO) or four (according to the American Psychiatric Association) primary symptom clusters: intrusive re-experiencing (e.g., flashbacks and nightmares), avoidance of trauma-related stimuli, hyperarousal symptoms (e.g., heightened startle response and insomnia), and negative alterations in cognition and mood [1,2]. PTSD is a significant global health burden, with a lifetime prevalence of 7–8% in the general population and much higher rates among trauma-exposed groups such as military personnel and motor vehicle accident (MVA) survivors [3,4]. While PTSD is commonly studied in isolation, it frequently co-occurs with traumatic brain injury (TBI), particularly in survivors of motor vehicle accidents (MVAs). This comorbidity introduces diagnostic and treatment challenges, as PTSD and TBI share overlapping symptoms, including cognitive impairment and emotional dysregulation.

**Figure 1:**
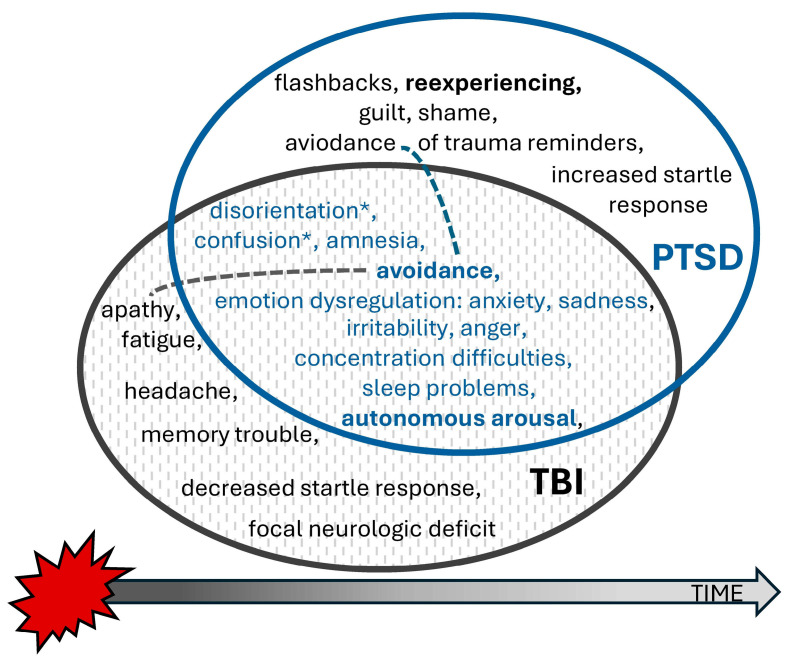
Overlap of post-concussion symptoms and PTSD influencing differential diagnosis. Disorientation and confusion, marked with asterisks, are characteristic of *acute stress reactions* (as such, they can be observed and also reported in people under extreme psychological stress without brain injuries).

Traumatic brain injury is defined as “an alteration in brain function, or other evidence of brain pathology, caused by an external force”, while an alteration in brain function is indicated by “any period of loss of or a decreased level of consciousness; any loss of memory for events immediately before (retrograde amnesia) or after the injury; neurologic deficits (weakness, loss of balance, change in vision, dyspraxia paresis/plegia [paralysis], sensory loss, aphasia, etc.); any alteration in mental state at the time of the injury (confusion, disorientation, slowed thinking, etc.)” [5]. The literature usually distinguishes between three levels of brain injury severity. TBI severity—classified as mild, moderate, or severe—introduces distinct challenges, including cognitive impairments, emotional dysregulation, and neurophysiological changes that may affect therapy outcomes (Mild brain injury refers to when no structural changes are observed in the brain, the Glasgow Coma Scale (GCS) score is 13–15, the person remains unconscious for no more than 30 min, and post-traumatic amnesia lasts no longer than 24 h. In the case of moderate brain injury, GCS scores are between 9 and 12 points, the person may be unconscious for less than 24 h, amnesia may persist for less than 7 days, and changes in brain structure may be observed in neuroimaging tests. To diagnose severe brain injury, the score on the GCS must be below nine, the individual must be unconscious for more than 24 h, the post-traumatic amnesia must extend beyond 7 days, and neuroimaging may show abnormalities in brain structure [5]) [6]. Mild TBI (mTBI), commonly referred to as a concussion, typically results in transient neurological symptoms. Although many patients fully recover from mild-to-moderate brain injuries within a few days, in some of these patients, the duration of symptoms may be prolonged, constituting so-called post-concussion syndrome (PCS). Its main symptoms usually belong to four categories: cognitive deficits (e.g., disorders of concentration, memory, and executive functions), physical symptoms (headaches and dizziness, fatigue, nausea, hypersensitivity to light and noise, and sleep disorders), psychiatric symptoms (anxiety, depression, frustration, and excessive avoidance), and emotional dysregulation (e.g., irritability and emotional lability) [7] (see Figure 1). Little is known about the impact of *traumatic brain injury* *as a comorbid condition on treatment outcomes*, although TBI often presents overlapping symptoms, potentially complicating PTSD diagnosis, treatment planning, and outcome (Figure 1).

A full recovery from PCS usually takes no longer than three months. However, 7–33% of all individuals experiencing traumatic injury exhibit the above symptoms chronically, significantly affecting their functioning [5]. Much research on PTSD and TBI has focused on military personnel in conflict settings. Less is known about the prevalence rates and treatments of PTSD in civilian TBI, which is typically caused by a motor vehicle accident, an assault, a sports injury, or a fall. Even if a general trend indicates an increase in safety on the roads in Europe, there is still a significant number of car accident participants who experienced TBIs, suffer from PTSD, and might benefit from appropriate evidence-based treatment [4].

Treatments of PTSD, namely psychological and pharmacological treatments, have an empirical background reflected in health technology agency (HTA) assessments and guidelines (like the National Institute for Care and Clinical Excellence (NICE)). Trauma-focused, evidence-based cognitive-behavioral therapies (CBTs) like prolonged exposure (PE) and cognitive processing therapy (CPT) are recommended for PTSD as a first-line treatment (NICE, APA) [6,8]. The literature on treatments under the umbrella name “non-trauma-focused treatments” is limited, but such trials on PTSD are important, especially for individuals with comorbid TBIs who may find trauma-focused approaches challenging.

However, individuals with co-occurring TBIs are often excluded from clinical trials. As such, there are limited data on the efficacy of these interventions for PTSD + TBI populations, especially for civilian trauma like motor vehicle accidents and interpersonal violence survivors, which makes this a growing civilian health challenge [4].

The available studies on treatments of PTSD in patients who experienced TBIs still share a common conclusion: a “need for further studies” [9,10]. The key findings suggest that trauma-focused cognitive behavioral therapies such as PE and cognitive processing therapy (CPT) are effective even in individuals with TBIs [6,11,12]. Moreover, systematic reviews (e.g., [10,13]) confirmed that such interventions are well tolerated in mild TBI populations when appropriately adapted. However, data from real-world studies suggest that while psychotherapy is effective for mTBI, dropout rates are higher compared with PTSD-only populations due to cognitive fatigue and somatic symptoms [13]. Ambiguity also remains regarding the impact of cognitive deficits on treatment response. While Rauch et al. found no reduction in tolerability [14], other sources (e.g., [13]) suggest that impairments in memory or executive function may limit the applicability of exposure-based protocols in more severe TBI. The findings of Rauch et al. [14] provide strong support for the use of prolonged exposure (PE) therapy in veterans with PTSD and comorbid mild-to-moderate traumatic brain injuries (TBIs). The study demonstrated that PE led to significantly greater reductions in PTSD symptoms and higher rates of remission compared with present-centered therapy (PCT), a non-CBT and non-trauma-focused approach, with no difference in treatment retention or tolerability.

Specifically, participants who received PE showed a mean reduction of 22.2 points on CAPS-5, compared with 14.7 points in the PCT group. Moreover, 45.3% of the PE group no longer met the criteria for PTSD post treatment, compared with 27.7% in the PCT group. The rates of reliable clinical improvement (defined as a reduction ≥ 13 points on CAPS-5) were also higher in the PE group (64.1%) than in the PCT group (45.3%), although this difference reached only borderline statistical significance. Importantly, both treatments were well tolerated, with comparable rates of session attendance and retention.

The idea for a future clinical trial comparing prolonged exposure (PE) with self-efficacy-focused cognitive therapy (SEF-CT) emerged from our earlier studies [15,16], in which a small yet meaningful number of participants either declined to enroll in PE or discontinued treatment due to the trauma-focused nature of the intervention. This observation underscored the need to evaluate alternative approaches that may be more acceptable to certain subgroups of patients with PTSD. Individuals with PTSD and co-occurring TBIs create one such subgroup. In particular, it remains unclear whether trauma-focused interventions (e.g., prolonged exposure) or non-trauma-focused therapies (e.g., SEF-CT) are equally effective for this (civilian) population. This secondary analysis of PTSD treatment outcomes focused on the role of comorbidity (TBI). This study addresses this gap by comparing treatment outcomes between PTSD + TBI and PTSD-only participants receiving either PE or SEF-CT.

This study aimed to evaluate the effectiveness of PTSD treatments (psychotherapy) in individuals with TBIs, focusing on treatment response and the role of time elapsed since the MVA. Experiencing TBI (H1) was expected to predict a smaller recovery rate than for non-TBI subjects. Considering the role of the time after an MVA and the neurological effects of TBI, it was expected that a lesser effect would occur in a shorter time (H2). Although the data from the meta-analysis suggest no effect from the time after MVA and entering therapy [17], our earlier study, as well as other authors’ results, indicate that time is a substantial moderator of therapy effectiveness [18,19].

The participants were MVA victims, representing the largest homogenous group of PTSD patients in Poland. The efficacy of trauma-focused CBT in treating PTSD resulting from car accidents has previously been demonstrated [16,18,19,20,21,22]. The present study used archival clinical data of the research program TRAKT-3 on PTSD treatments to assess the role of comorbid TBI on the effectiveness of prolonged exposure (PE) and a non-trauma self-efficacy-focused cognitive therapy (SEF-CT) for post-traumatic stress disorder (PTSD) among MVA victims. The study was registered at the National Science Centre (2012/06/A/HS6/00340), and all procedures including informed consent to participate in a clinical trial were accepted by the local IRB at the University of Warsaw [16].

## 2. Materials and Methods

### 2.1. Participants

This study included MVA survivors diagnosed with PTSD being predominant in the clinical picture, based on DSM-5 criteria, participating in a clinical trial on the efficacy of PE and SEF-CT in PTSD treatment. Exclusion criteria were limited to (1) elevated suicide risk or unstable medical conditions, (2) co-occurring psychiatric disorders requiring changes in psychotropic medication, (3) non-adherence to study protocols (e.g., refusal of random allocation or failure to attend therapy sessions), and (4) severe neuropsychological deficits (e.g., aphasia, apraxia) precluding participation in talk therapy. Out of 152 participants (The primary goal of this study was to compare the effectiveness of SEF-CT and PE. The required sample size was estimated to be 148 subjects using the GPower 3.1 program for power = 0.95 (1 − β error) and alpha = 0.05 for repeated-measures ANOVA, with a low effect size (0.30) and two assessment points (pre- and post-treatment)) who qualified for the therapy, 18 (11.8%) dropped out (7 out of 76 from CP (9.2%) and 11 out of 76 from PE (14.5%)). In the overall ITT sample, 53 patients reported TBIs (34.9%) and did not differ in either treatment arm (*Χ*^2^(1, *N* = 152) = 3.51, *p* = 0.06). The dropout rate did not differ between therapies (*Χ*^2^(1, *N* = 152) = 1.01, *p* = 0.32) or between TBI and non-TBI subjects (8/53 (15.1%) and 10/99 (10.1%), respectively) (*Χ*^2^(1, *N* = 152) = 0.82, *p* = 0.36). Dropout cases did not differ from those who completed therapy with regard to demographic characteristics (gender, age, and education) or time from the MVA, PTSD symptoms, or intelligence tests results. The same findings were obtained within the subgroup of TBI subjects as well as the non-TBI subsample. Due to the non-significant differences between psychotherapies as well as subjects with and without TBIs, dropout cases were not taken into account, and thus the final sample was based on those who completed therapy (*N* = 134; 65 for PE versus 69 for CT) and for whom the assessment of post-treatment PTSD was obtained. The analyses showed that both treatment groups did not differ concerning any variable (except gender, a difference not recorded in the ITT sample). Their demographic characteristics and the overall sample are presented in Table 1.

### 2.2. Psychotherapy

Two kinds of cognitive behavioral psychotherapy were applied. A protocol for a non-trauma-oriented *self-efficacy-focused cognitive therapy for PTSD* (SEF-CT) was designed based on research regarding the structure of PTSD symptoms [20,21] and research concerning the underlying processes that contribute to the maintenance of PTSD symptoms. Beginning with individual conceptualization and goal setting, participants were engaged in modules involving techniques like psychoeducation, cognitive restructuring, arousal reduction, behavioral activation, coping and emotion regulation, and problem solving [22]. Traumatic memories were not addressed during the treatment. *Prolonged exposure therapy* (PE), developed by Edna Foa et al. [23], is a well-established trauma-focused treatment [5,6,7] composed of psychoeducation, in vivo and prolonged imaginal exposure, breathing exercises, and processing.

Both treatments consist of 10 90-min weekly sessions delivered over three months by a psychologist trained according to EABCT standards in cognitive behavioral therapy (CBT) [24], as well as both treatment protocols.

### 2.3. Instruments

The diagnosis of PTSD according to DSM-5 [1] was established by a psychiatrist independent from the therapy condition, the staff of psychiatrists in the Polish CAPS-5 interview translations [25,26], and the self-reporting PDS-5 inventory translations [20,27]. Both instruments enabled the assessment of PTSD symptoms lasting more than one month (20 items with a 5-point Likert scale, whose scores were dichotomized into symptoms and where in both instruments, all assessments were recoded into symptoms). Both measures were applied before and after the treatment in this sample. The analyses demonstrated high reliability (*α* = 0.93 for CSPS-5 and 0.92 for PDS-5 for 20 symptoms) and high congruency between diagnoses for both measures (89.6% consistent diagnoses; Cohen’s *kappa* = 0.79).

In this study, the demographic variables were also recorded (gender, age, education, and time after the MVA), as well as comorbid psychiatric disorders identified via mental status examination, with a clinical interview in addition to CAPS-5.

Traumatic brain injury (TBI) was conceptualized as experiencing head injury during an accident with loss of consciousness for any duration, taking into consideration the possible overlap of PCS with PTSD, as indicated in Figure 1. Participants were assessed for TBI as a part of a comprehensive mental state examination by a psychiatrist blind to the treatment conditions, who recorded answers to the questions about head trauma during MVAs associated with loss of consciousness (LOC) and the estimated times of LOC. Inclusion into the TBI group was based on the reported loss of consciousness and post-traumatic amnesia related to accidents, supported by presenting documents from the emergency department where the patient was treated after their accident.

In the studied sample, 33.6% of the subjects (45 subjects out of 134) reported TBIs during an accident, causing post-traumatic amnesia and lasting from several minutes to days (what may be classified as mild-to-severe injuries) [5]. Before psychotherapy, possible mental impairment was assessed using the experimental version of Cattell’s and Weiβ’s *culture-free test* (CFT) (Polish adaptation: Stańczak [28]) as a marker of fluid intelligence (26 nonverbal items with a time limit of 18 min) and an experimental form of the *vocabulary* test (synonyms) based on the APIS battery (14 items, with a time limit of 7 min) as a marker of crystallized intelligence [29].

Participants reporting TBIs did not differ from the non-TBI MVA participants concerning gender (*Χ*^2^(1, 134) = 0.05, *p* = 0.82), education (*Χ*^2^(1, 134) = 0.97, *p* = 0.33), age (*t* = (132) = 1.90, *p* = 0.06), time after the MVA (*t*(132) = 1.83, *p* = 0.07), the pre-treatment number of PTSD symptoms assessed by CAPS-5 (*t*(132) = 0.07, *p* = 0.84) or PDS-5 (*t*(132) = 0.23, *p* = 0.82), the post-treatment PTSD symptoms assessed by CAPS-5 (*t*(132) = 1.84, *p* = 0.07) or PDS-5 (*t*(132) = 0.68, *p* = 0.50), or the diagnosis of PTSD by CAPS-5 (*Χ*^2^(1, 134) = 1.59, *p* = 0.21) or PDS-5 (*Χ*^2^(1, 134) = 0.78, *p* = 0.38). Among the participants who reported TBIs, 24.4% (11 out of 45) experienced severe brain injuries, with other subjects experiencing mild traumatic injuries. The differences between both subgroups were not significant concerning gender (*Χ*^2^(1, 45) = 1.14, *p* = 0.28), education (*Χ*^2^(1, 45) = 0.18, *p* = 0.67), age (*t*(1, 43) = 1.88, *p* = 0.07), time after the MVA (*t*(1, 43) = 0.47, *p* = 0.64), the pre-treatment number of PTSD symptoms assessed by CAPS-5 (*t*(1, 43) = 0.69, *p* = 0.50) or PDS-5 (*t*(1, 43) = 0.07, *p* = 0.95), the post-treatment PTSD symptoms assessed by CAPS-5 (*t*(1, 43) = 1.84, *p* = 0.07) or PDS-5 (*t*(1, 43) = 0.41, *p* = 0.69), or the diagnosis of PTSD by CAPS-5 (*Χ*^2^(1, 45) = 0.23, *p* = 0.63) or PDS-5 (*Χ*^2^(1, 45) = 0.06, *p* = 0.80). Due to the findings, both subgroups were combined into one TBI sample.

### 2.4. The Procedure of Data Analysis

Analysis of covariance (ANCOVA) for post-treatment PTSD symptoms was performed, with the pretreatment symptoms controlled independently for both instruments. The model included the main effects of gender, age, education (dichotomized (university level) versus others (college, job, school, etc.)), therapy (PE versus SEF-CT), time after the MVA (due to the skewness of distribution, median split was ≤12 months versus >12 months), TBI during the MVA (present versus absent), and level of fluid intelligence. (Due to the significant association of the CFT test scores with brain injuries, only the fluid intelligence indicator (not the results of the vocabulary test) was taken into account.) Considering the expected power of the model, all two-way interactions were calculated only for TBI. Due to the non-significant effect of almost all independent variables, the final model was trimmed to the time after the MVA and TBI, with the control of the pretreatment PTSD level. In the next step, the correlational analyses (Person’s *r* and *Eta*) were applied to the overall sample as well as the subsamples, distinguished on the basis of TBI and time after the MVA. All statistical analyses were performed using SPSS 29.0, which also provided the graphical forms of the tested effects.

## 3. Results

### 3.1. Treatment Effectiveness for PTSD Symptoms After Psychotherapy in TBI and Non-TBI Participants: Clinical Improvement

Following Kazdin’s [30] framework for clinically significant change, one key indicator is remission, defined as the proportion of patients who no longer meet the diagnostic criteria for the disorder post treatment. In this study, the CAPS-5-based remission rate (i.e., not meeting criteria for PTSD post treatment) was as follows. Among the participants with PTSD only, remission was observed in 95.8 and 90.2% of cases for the shorter and longer treatment durations, respectively. For the participants with comorbid PTSD and TBI, the remission rates were 77.8% (shorter duration) and 92.6% (longer duration).

Reliable clinical improvement (RCI) on CAPS-5 was calculated using the reliable change index method described by Jacobson and Truax [31]. Based on existing research [26], a reduction of ≥13 points in the total CAPS-5 severity score is a widely accepted threshold for reliable improvement, though some studies use a more conservative cutoff of ≥10 points. In the current sample of treatment completers, 91.0% achieved reliable improvement (RCI ≥ 13). Among the non-TBI participants, the RCI rates were 92.1% overall, with 95.8% for the shorter-duration group and 87.8% for the longer one. Among the TBI participants, the overall RCI was 88.9%, with 83.3 and 92.6% in the shorter- and longer-duration groups, respectively.

### 3.2. Treatment Effectiveness for PTSD Symptoms After Psychotherapy in TBI and Non-TBI Participants: ANCOVA

Across both instruments, analyses showed the significant effect of the pretreatment symptoms of PTSD. Non-significant main effects were found for therapy, gender, age, education, time after the MVA, and fluid intelligence level. Contrary to the expectation (which did not confirm H1), the main effect of TBI was also not significant. The interactions of TBI with other variables were also not significant (replicated by analyses of the results obtained for both instruments), which suggests that psychotherapy was equally effective for patients reporting brain injuries and the controls, regardless of gender, age, education, intelligence level, and psychotherapy mode. The only significant interaction was found for TBI and time after the MVA (which confirmed, in part, H2; see Table 2).

**Figure 2:**
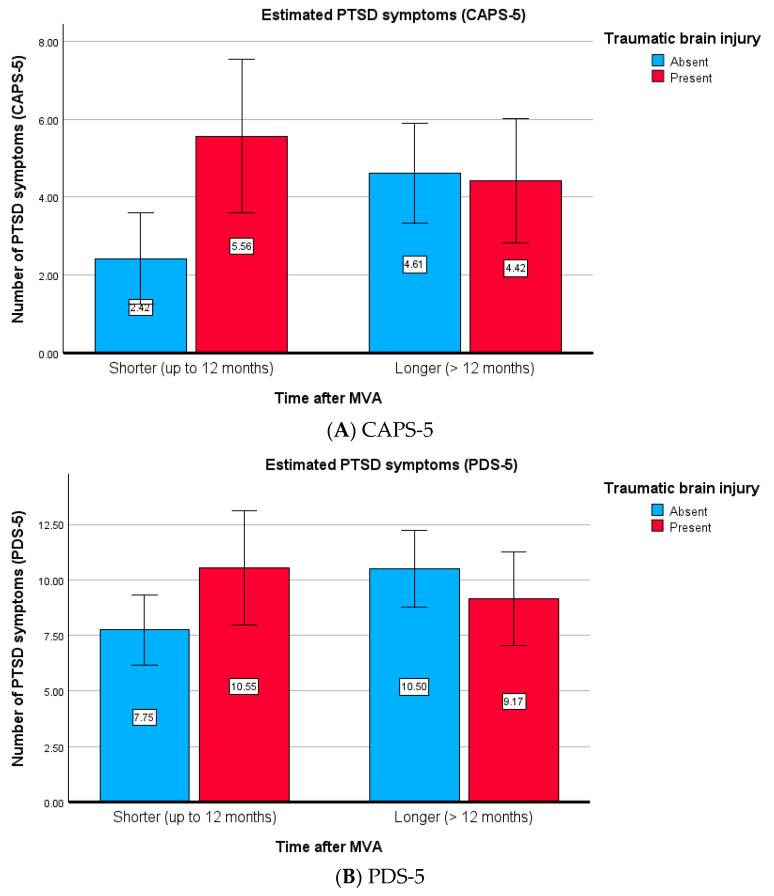
(**A**,**B**) Estimated means of post-treatment PTSD symptoms of the interaction of traumatic brain injury (absent vs. present) and the time after an MVA (shorter vs. longer). Note: TBI = absent or present reported brain injury during an MVA, with a shorter time (up to 12 months) or longer time after the MVA (>12 months). The 95% confidence intervals were drafted for each assessment. The rate of post-treatment PTSD remission (lack of PTSD diagnosis comprising criteria F and G in DSM-5) for CAPS-5 was as follows: 95.8 and 90.2% for shorter and longer times for absent TBI, respectively, and 77.8 and 92.6% for present TBI, respectively. The rate of post-treatment PTSD diagnosis for PDS-5 was as follows: 89.6 and 73.2% for shorter and longer times for absent TBI, respectively, and 72.2% and 77.8% for present TBI, respectively.

This interaction is graphically depicted in Figure 2A,B. Post hoc contrast tests showed that a significant difference in post-treatment PTSD symptoms was found only between the non-TBI subjects (CAPS-5: *F*(1, 129) = 6.235, *p* = 0.014, *η*^2^ = 0.046; PDS-5: *F*(1, 129) = 5.456, *p* = 0.021, *η*^2^ = 0.041), with no significant effects observed among the TBI patients (CAPS-5: *F*(1, 129) = 0.774, *p* = 0.380, *η*^2^ = 0.006; PDS-5: *F*(1, 129) = 0.668, *p* = 0.415, *η*^2^ = 0.005). The difference between the TBI and non-TBI subjects was also significant for shorter times (CAPS-5: *F*(1, 129) = 7.280, *p* = 0.008, *η*^2^ = 0.053; PDS-5: *F*(1, 129) = 3.336, *p* = 0.070, *η*^2^ = 0.025) but not for longer times after MVAs (CAPS-5: *F*(1, 129) = 0.036, *p* = 0.849, *η*^2^ = 0.000; PDS-5: *F*(1, 129) = 0.945, *p* = 0.333, *η*^2^ = 0.007). The data suggest that therapy is less effective for non-TBI subjects who begin treatment later compared with those who start earlier. By contrast, for the TBI subjects, the treatment was equally effective regardless of the time elapsed after the MVAs.

In summary, the results indicate that in non-TBI subjects, a longer time between the MVA and the start of treatment reduced treatment effectiveness (with higher effectiveness observed for earlier treatment). The TBI subjects did not benefit from an earlier start in therapy, demonstrating similar overall effectiveness. This also suggests that the differences between TBI and non-TBI subjects may be more apparent in the early period after an MVA and may be related to the characteristics of TBI subjects who enter treatment sooner.

### 3.3. Intelligence Level and Other Variables in TBI and Non-TBI Participants: Comparisons of Subgroup Differences

Following this idea, we examined the results of all demographic variables and intelligence across all subgroups. Notably, subjects who reported TBIs but entered therapy earlier, compared with those who started later, did not differ in terms of TBI severity (*Χ*^2^(1, 45) = 0.98, *p* = 0.32), gender (*Χ*^2^(1, 45) = 1.10, *p* = 0.29), education (*Χ*^2^(1, 45) = 0.02, *p* = 0.90), or age (*t*(1, 43) = 0.66, *p* = 0.51), nor did they differ from the participants without TBIs. However, the TBI patients showed a lower level of fluid intelligence (*t*(1, 43) = 1.98, *p* = 0.05; *M* = 14.43 versus 17.58). Such an effect was not found for the non-TBI subjects or the vocabulary test. No differences were found in treatment allocation. For the shorter time, the TBI and non-TBI groups differed concerning fluid intelligence (*t*(1, 64) = 2.70, *p* = 0.01; *M* = 14.43 versus 18.62) but not for the vocabulary test. A difference was also found for age (*t*(1, 64) = 2.31, *p* = 0.03; TBI subjects were older), with no differences for gender (*Χ*^2^(1, 66) = 0.01, *p* = 0.98) or education (*Χ*^2^(1, 66) = 0.86, *p* = 0.35). No differences were found for the longer-time groups or treatment allocation. In the next step, the correlations between the intelligence tests and TBI as well as PTSD were calculated in the overall sample as well as in subsamples distinguished on the basis of time and TBI.

### 3.4. Intelligence and Symptoms of PTSD in TBI and Non-TBI Subjects: Correlational Analyses

The association between brain injury severity and the results of the intelligence tests was not significant (*N* = 45, *Eta* = 0.11 and 0.021). However, a significant correlation was found between the reported brain injury—regardless of its severity—and performance for the culture-free test (CFT, assessing fluid intelligence), where *Eta* = −0.20 (*N* = 134, *p* = 0.02). The sign of the *Eta* correlation was added after analysis of the group differences (groups were coded as lack of TBI = −1 and TBI = 1). No significant correlation was found for the vocabulary test (*N* = 134, *Eta* = −0.16, *p* = 0.06), although vocabulary scores were significantly correlated with the education level (*Eta* = 0.19, *p* = 0.02). However, a significant correlation between TBI and fluid intelligence was observed only in subjects assessed shortly after an MVA (*Eta* = −0.32; *N* = 66; *p* = 0.01) and not for the vocabulary test (*Eta* = −0.11). No such correlation was found among the subjects who entered therapy later (*Eta* = −0.11 and 0.21, respectively). These findings suggest that individuals reporting TBIs following MVAs may experience minor cognitive impairment, as indicated by slightly poorer performance on the fluid intelligence test, particularly in the early post-accident period.

Considering the symptoms’ categories, significant correlations were found in the shorter post-MVA period for CAPS-5 baseline symptom intensity (calculated as the sum of items’ scores to obtain continuous results) and the CFT scores (category B: *r* = −0.28, *p* = 0.03; category C: *r* = −0.28, *p* = 0.03; category D: *r* = −0.24, *p* = 0.06; category E: *r* = −0.22, *p* = 0.08), similar to the post-therapy CAPS-5 assessment. No significant correlations were found for the PDS-5 (baseline, post-treatment) scores. The stepwise linear regression indicated that the intensity of symptoms for category C (avoidance) assessed by CAPS-5 was linked to fluid intelligence test performance only in the earlier post-MVA period.

## 4. Discussion

The findings of this study support the conclusion that individuals with PTSD and comorbid TBIs following motor vehicle accidents (MVAs) can respond favorably to cognitive behavioral therapy (CBT), with the overall treatment outcomes comparable to those for individuals without TBIs. Both PE and SEF-CT produced clinically meaningful improvements in PTSD symptoms across the groups, though the PTSD-only participants exhibited more rapid gains in the short term.

The observed effectiveness of PE aligns with existing research supporting the efficacy of trauma-focused treatments for individuals with PTSD and TBIs [9]. By contrast, the effectiveness of SEF-CT for PTSD + TBI patients represents a novel contribution, as does the overall efficacy of this non-trauma-focused approach. While PE remains a first-line treatment, SEF-CT was specifically designed in part to address limitations observed in trauma-focused therapies [16]. SEF-CT integrates core CBT principles [22] with strategies emphasized by the Lancet Neurology Commission, namely self-perception reappraisal, problem-focused coping, and enhancement in self-efficacy [32].

Despite similar endpoint outcomes, the distinct hypothesized therapeutic mechanisms of PE (exposure-based) and SEF-CT (cognitive and formulation-driven) underscore the importance of matching intervention approaches to individual needs. Notably, an earlier initiation of therapy was associated with lower baseline fluid intelligence in the PTSD + TBI group, which may reflect the cognitive effects of TBIs. These impairments may interfere with key therapeutic processes such as emotional processing, trauma-related memory integration, and the generalization of learned skills. Consequently, delayed or attenuated treatment response in this group may relate to cognitive challenges that moderate therapy efficacy. These considerations are further supported by analyses which demonstrated high rates of reliable clinical improvement and PTSD remission across subgroups. Importantly, individuals with TBIs responded similarly to those without them, suggesting that cognitive impairments do not preclude treatment success when protocols are adapted accordingly.

Two additional findings concerning time and intelligence warrant further discussion. The relationship between PTSD, TBI, and treatment outcomes involves multiple factors, including the timing of treatment initiation and symptomatology (see Figure 1). “Any loss of memory” is the main criterion of TBI [5], but memory impairments commonly associated with acute stress disorder or PTSD may contribute to partial amnesia surrounding the injury event. Patients with preserved memory of the accident but amnesia for events occurring afterward may experience this memory disruption due to stress or medications administered post injury. Although research has demonstrated a neural dissociation between PTSD and TBI [33], clinically differentiating these conditions remains challenging. For instance, as illustrated in Figure 1, avoidance behavior may stem from the avoidance of memories and trauma reminders in PTSD or from pain and apathy in patients with post-concussive syndrome (PCS). Considering that TBI often has a minimal long-term impact on neuropsychological functioning, PTSD appears to be the primary factor contributing to cognitive deficits in these patients. The natural course of PCS complicates distinguishing how much PTSD symptomatology is influenced by TBI in the early post-accident phase.

A notable finding of this study is that PTSD + TBI patients who began treatment earlier exhibited slightly lower fluid intelligence scores at the pretreatment assessment. This may reflect cognitive impairments typically associated with TBI, such as reduced problem-solving ability, abstract reasoning, and executive functioning [33,34,35]. These deficits could limit patients’ ability to fully engage in and benefit from learning-based psychotherapies during the early post-accident period. For example, PE requires active recall and processing of traumatic memories, along with the application of new coping strategies, while SEF-CT emphasizes cognitive restructuring and problem solving. Both treatments aim to enhance emotional regulation skills, but deficits in abstract reasoning and working memory may hinder patients’ ability to interpret their trauma, recognize avoidance patterns, or generalize therapeutic gains to new contexts. Furthermore, lower cognitive performance may impair emotional regulation, a crucial component of psychotherapy. As a result, PTSD + TBI patients may struggle with maintaining focus during therapy sessions or consolidating the emotional processing of traumatic events, leading to slower or less sustained symptom improvement. These findings may explain the time-related observation that PTSD-only patients benefitted more from early treatment initiation, whereas PTSD + TBI patients showed a pattern of recovery characteristic of PTSD-only participants starting treatment a longer time after their MVAs, likely due to additional PCS-related cognitive symptoms. Given that both applied psychological treatments are based on learning, the early initiation of treatment for PTSD-only patients gives them the opportunity to integrate new knowledge against PTSD-maintaining mechanisms such as avoidance, beliefs about danger, and impaired self-efficacy. TBI-related symptoms may handicap this “time advantage” in PTSD recovery. The results suggest that cognitive impairments act as a moderating factor in the effectiveness of psychotherapy for PTSD + TBI patients. Addressing these challenges by adapting treatment protocols—for instance, by incorporating breaks and frequent repetition of key information—could help mitigate the impact of cognitive deficits.

Two clinical case examples from the TRAKT study illustrate the feasibility of personalized treatment matching. One participant with a mild TBI and severe psychosocial disruption experienced substantial recovery following PE. Another with a severe TBI and significant cognitive sequelae achieved full remission with SEF-CT. Both individuals expressed validation and relief upon receiving a PTSD diagnosis and accessing targeted treatment. These cases exemplify the value of patient-centered care models that acknowledge cognitive status, symptom profiles, and therapeutic alignments.

Taken together, these findings reinforce the importance of expanding PTSD treatment options while ensuring each is anchored in a mechanistic understanding of PTSD maintenance and cognitive behavioral principles. The aim is not to dilute evidence-based standards but to enhance reach, precision, and effectiveness in complex clinical populations.

This study has several *limitations*. While focused on PTSD treatment efficacy, TBI was considered a possible moderator, but its assessment was limited to pretreatment anamnesis data. A lack of formal TBI assessment and neurophysiological diagnosis clearly limits the conclusions of this study. Another limitation of this study lies in the demographic composition of the sample, which was predominantly composed of mid-life women. This homogeneity restricts the generalizability of the findings to broader populations. Age-related differences may significantly influence the expression of PTSD symptoms as well as the responsiveness to cognitive behavioral therapy (CBT), with younger individuals potentially exhibiting greater neuroplasticity and older adults facing additional neurocognitive challenges that may affect treatment outcomes. Similarly, the overrepresentation of women limits the applicability of the results to men and nonbinary individuals, who may have different trauma exposures and patterns of psychological response. Furthermore, the study sample primarily included individuals exposed to motor vehicle accidents, which may not reflect the complexity of PTSD associated with other trauma types such as combat, childhood abuse, or sexual violence. These different trauma contexts can interact uniquely with traumatic brain injury (TBI), potentially altering both symptomatology and the efficacy of CBT. The lack of ethnic, cultural, and socioeconomic diversity further narrows the interpretability of the results, as cultural factors and access to care can substantially affect engagement with and the response to psychotherapy. To enhance the generalizability and clinical utility of the findings, future research should aim to include more demographically diverse samples with varied age ranges, gender identities, trauma histories, and sociocultural backgrounds. *The clinical implications* can be summarized as follows:

- Patients with PTSD and comorbid TBIs can benefit substantially from psychological treatment, with outcomes comparable to those without TBIs.
- Both PE and SEF-CT can be effective. However, individual differences in cognitive functioning, timing of intervention, and treatment preferences should guide therapy selection.
- The presence of a TBI may delay the benefit of psychotherapy due to cognitive symptoms such as reduced fluid intelligence or executive dysfunction.
- Personalized treatment protocols that account for cognitive limitations—such as slower pacing, repetition, and memory support—may enhance outcomes in TBI populations.
- The accurate diagnosis of PTSD in the context of TBI is essential. The misattribution of symptoms to “organic” injury alone may hinder access to effective, targeted interventions.
- Expanding therapeutic options beyond trauma-focused modalities can improve engagement and outcomes, particularly for patients reluctant or unable to participate in exposure-based treatments.
- Clinicians should consider both the clinical profile and patient preference when recommending therapy, ensuring alignment between the intervention’s demands and the patient’s cognitive–emotional capacities.
- Importantly, expanding treatment options should not imply non-specific or non-mechanistic interventions. Each therapeutic approach must be guided by a clear treatment rationale and grounded in established models of PTSD maintenance mechanisms. Manualized protocols such as SEF-CT should be explicitly linked to conceptual frameworks and supported by emerging empirical evidence to ensure clinical fidelity and theoretical consistency.

The following are clinical case examples.

Patient 1: Woman, 48 years old, married, two sons (23 and 11). Before an accident, she was a university lecturer in the field of chemistry. Accident: A year before admission to the program, she was hit by a car when crossing the street with her 11-year-old son. Her son spent several weeks in critical condition, and the patient had several hours of LOC. Upon admission, the patient complained of headaches, lack of hand coordination, memory problems, sleep disorders, and depression. At admission to the program, the DSM-5 criteria for PTSD were met. No personality disorders were present. In anamnesis and documentation, MRI showed two small vascular lesions (3 mm) in the periventricular white matter of the right hemisphere of the brain. No focal changes or process features were present. Severe impairment of social and professional (disability pension) functioning were observed, Ongoing court proceedings took place.

This patient was randomized to receive 10 sessions of prolonged exposure. Post-treatment assessment showed no PTSD, improvement in overall functioning and QOL while actively job searching and having active social and family lives.

Patient 2: Woman, 35 years old, higher education (Ed.D.), free marital status, 10 years of professional activity in a highly competitive corporation. Accident: A year before admission to the program, the patient was in a car crash, resulting in the death of her father. She suffered a head injury and fractures in the upper and lower limbs, spent 2 weeks in a coma, and had a severe TBI. At admission, she presented PTSD and adjustment disorder. A personality change due to a known physiological condition (“organic personality”) was shown.

Randomized to receive 10 sessions of SEF-CT. Post-treatment assessment: no PTSD, improvement in overall functioning and QOL, return to social life, work, and romantic relationship.

Both patients expressed the following:

“Looking for help I visited several doctors, many times describing my symptoms. I heard, so many times, that my condition was “normal” after such an accident, I should accept that it was “organic”. It only deepened my sadness and the feeling of how far away I was from my previous life. It was amazing in the program, that just hearing the questions at the PTSD diagnosis, I had the impression that I finally came to a place where both sides were thinking/talking about the same. Thank you.”

## 5. Conclusions

Traumatic brain injury is a common experience among individuals who develop PTSD as a result of combat exposure, blasts, motor vehicle accidents, or assaults. Focusing on a single aspect of this comorbidity may delay effective treatment interventions.

This study contributes to the existing literature [36,37,38,39,40] by demonstrating that patients who sustain a TBI during a car accident and subsequently develop PTSD can benefit from both prolonged exposure therapy (a trauma-focused treatment) and SEF-CT (a non-trauma-focused, self-efficacy-oriented cognitive therapy for PTSD). While prolonged exposure remains the gold standard for PTSD treatment, the promising results of SEF-CT warrant further replication in future research.

## Figures and Tables

**Table 1:** Demographic characteristics of subjects participating in psychotherapy.

Sample	Time After MVA *M* (*SD*)	Education: University vs. Other (Percent)	Traumatic Brain Injury (Percent)	*N*	Gender	Age (Range)	Age *M* (*SD*)
PE	21.14 (19.76)	38/27 (58.5%)	27 (41.5%)	65	39F/26M	22–68	39.97 (12.01)
SEF-CT	18.28 (17.61)	50/19 (72.5%)	18 (26.1%)	69	55F/14M	18–67	36.72 (10.40)
Total	19.68 (18.67)	88/46 (65.7%)	45 (33.6%)	134	94F/40M	22–68	38.30 (11.34)

Note: PE =prolonged exposure; SEF-CT = self-efficacy-focused cognitive therapy. Differences between therapy groups with regard to gender (*Χ*^2^(1, 134) = 6.21, *p* = 0.02), education (*Χ*^2^(1, 134) = 2.91, *p* = 0.09), number of subjects reporting brain injuries (*Χ*^2^(1, 134) = 3.58, *p* = 0.06), age (*t*(132) = 1.67, *p* = 0.10), time after MVA (*t*(132) = 0.88, *p* = 0.38), pre-treatment number of PTSD symptoms assessed by CAPS-5 (*t*(132) = 0.73, *p* = 0.47) and PDS-5 (*t*(132) = 0.08, *p* = 0.93), post-treatment PTSD symptoms assessed by CAPS-5 (*t*(132) = 0.72, *p* = 0.48) and PDS-5 (*t*(132) = 1.09, *p* = 0.48), and diagnosis of PTSD by CAPS-5 (*Χ*^2^(1, 134) = 0.25, *p* = 0.62) and PDS-5 (*Χ*^2^(1, 134) = 0.22, *p* = 0.64).

**Table 2:** Results of ANCOVA for symptoms of PTSD assessed after psychotherapy.

Instrument	CAPS-5			PDS-5		
**Initial Model**	***F***	***p***	***η*** ^**2**^	***F***	***p***	***η*** ^**2**^
Corrected model	2.704	0.002	0.241 (0.152)	1.707	0.063	0.167 (0.069)
Constant term	0.982	0.324	0.008	0.330	0.567	0.003
Therapy	2.256	0.136	0.019	1.992	0.161	0.016
Gender	0.283	0.595	0.002	0.125	0.724	0.001
Age	0.680	0.411	0.006	3.881	0.051	0.032
Education	5.073	0.026	0.041	0.409	0.524	0.003
Fluid intelligence (IQ)	0.078	0.780	0.001	0.308	0.580	0.003
Time after MVA (time)	0.100	0.752	0.001	0.185	0.668	0.002
Pretreatment PTSD symptoms	10.926	0.001	0.084	9.815	0.002	0.076
Traumatic brain injury (TBI)	3.590	0.061	0.029	1.712	0.193	0.014
TBI × therapy	0.018	0.893	0.000	0.009	0.925	0.000
TBI × gender	0.052	0.820	0.000	1.220	0.272	0.010
TBI × age	1.753	0.188	0.015	0.139	0.710	0.001
TBI × education	0.256	0.614	0.002	0.245	0.621	0.002
TBI × IQ	1.027	0.313	0.009	0.556	0.457	0.005
TBI × time	5.121	0.025	0.041	4.275	0.041	0.035
**Final (trimmed) model**	***F***	***p***	***η*** ^**2**^	***F***	***p***	***η*** ^**2**^
Corrected model	7.286	<0.001	0.184 (0.159)	3.969	0.005	0.110 (0.082)
Constant term	2.550	0.113	0.019	0.469	0.495	0.004
Pretreatment PTSD symptoms	14.440	<0.001	0.101	9.455	0.003	0.068
Traumatic brain injury (TBI)	3.684	0.057	0.028	0.506	0.478	0.004
Time after MVA (time)	0.447	0.505	0.003	0.446	0.506	0.003
TBI × time	4.479	0.036	0.034	4.035	0.047	0.030

Note: *F* = *F* test; *p* = level of significance; *η*^2^ = *partial Eta square* indicator of effect size (in parentheses for models = corrected *R*^2^). Here, *df* = 1/119 and 1/129 for all effects in the initial and final models, respectively. Significant effects (except the constant term) are marked in gray. In the final model, the effects on the interaction of pretreatment PTSD symptoms with TBI and time were also tested (for *df* = 1/127, with CAPS-5, *F* = 2.499, *p* = 0.116, *F* = 0.418, and *p* = 0.519, and with PDS-5, *F* = 0.691, *p* = 0.407, *F* = 0.002, and *p* = 0.968).

## Data Availability

Data is contained within the article.
